# *Plasmodium yoelii nigeriensis* (N67) Is a Robust Animal Model to Study Malaria Transmission by South American Anopheline Mosquitoes

**DOI:** 10.1371/journal.pone.0167178

**Published:** 2016-12-02

**Authors:** Alessandra S. Orfano, Ana Paula M. Duarte, Alvaro Molina-Cruz, Paulo F. Pimenta, Carolina Barillas-Mury

**Affiliations:** 1 Laboratory of Malaria and Vector Research, National Institute of Allergy and Infectious Diseases, National Institutes of Health, Rockville, Maryland, United States of America; 2 Laboratory of Medical Entomology, Centro de Pesquisas René Rachou, Fundação Oswaldo Cruz—FIOCRUZ, Belo Horizonte, Minas Gerais, Brazil; 3 Fundação de Medicina Tropical Dr. Heitor Vieira Dourado, Manaus, Amazonas, Brazil; Université Pierre et Marie Curie, FRANCE

## Abstract

Malaria is endemic in the American continent and the Amazonian rainforest is the region with the highest risk of transmission. However, the lack of suitable experimental models to infect malaria vectors from the Americas has limited the progress to understand the biology of transmission in this region. *Anopheles aquasalis*, a major vector in coastal areas of South America, was found to be highly refractory to infection with two strains of *Plasmodium falciparum* (NF54 and 7G8) and with *Plasmodium berghei* (mouse malaria), even when the microbiota was eliminated with antibiotics and oxidative stress was reduced with uric acid. In contrast, *An*. *aquasalis* females treated with antibiotics and uric acid are susceptible to infection with a second murine parasite, *Plasmodium yoelii nigeriensis* N67 (PyN67). *Anopheles albimanus*, one of the main malaria vectors in Central America, Southern Mexico and the Caribbean, was more susceptible to infection with PyN67 than *An*. *aquasalis*, even in the absence of any pre-treatment, but was still less susceptible than *Anopheles stephensi*. Disruption of the complement-like system in *An*. *albimanus* significantly enhanced PyN67 infection, indicating that the mosquito immune system is mounting effective antiplasmodial responses. PyN67 has the ability to infect a broad range of anophelines and is an excellent model to study malaria transmission by South American vectors.

## Introduction

Malaria, a parasitic disease transmitted by mosquitoes, has a major impact on global public health and threatens the economy of one third of the world’s population. In the Americas, 22 countries are affected. In 2015, 660,000 cases of malaria were estimated and 120 million people in the Americas live in areas at risk of malaria [1]. The Amazonian rainforest is the region with the highest risk of transmission, however, the malaria threat extends throughout the Northern regions of South America and across Central America, the Caribbean and Mexico [2]. In Brazil, malaria affects thousands of people every year accounting for 10% of all cases reported outside Africa [1].

*Anopheles* mosquitoes are the vectors of several *Plasmodium* species that cause malaria in humans, with *Plasmodium vivax* (71%) and *Plasmodium falciparum* (29%) being the most prevalent infections in the Americas [1]. Despite the relative abundance of malaria in the New World, little is known about the biology of the mosquito vectors in this region, when compared to the vast knowledge available for vectors from Africa and Asia. Anopheline mosquitoes diverged from culicines approximately 217 million years ago (MYA;) [3] in the Pangea supercontinent. The three major subgenera of *Anopheles* (*Anopheles*, *Cellia*, and *Nyssorhynchus)* that transmit malaria to humans exhibit differing geographical ranges. Malaria vectors in Africa and Asia belong to the subgenus *Cellia* or *Anopheles* and diverged from the subgenus *Nyssorhynchus* about 125–115 MYA [4] as the landmasses that would become South America and Africa drifted apart. The subgenus *Cellia* is restricted to the Old World, while *Nyssorhynchus* is limited to tropical regions of the New World [5]. Several malaria vectors of the subgenus *Kerteszia* are also present in South America (e.g. *An*. *cruzzi*, *An*. *bellator* and *An*. *neivai*).

The laboratory colonization of American vectors of the subgenus *Nyssorhynchus*, such as *An*. *aquasalis* and *An*. *albimanus*, opened the possibility of studying their interactions with *Plasmodium* parasites. *An*. *aquasalis* is an important vector in coastal areas of South America, and its colonization was achieved in 1995 [6]. This was followed by the colonization of *An*. *albimanus*, one of the main malaria vectors in Central America, Southern Mexico and the Caribbean [7]. *An*. *aquasalis* and *An*. *albimanus* are the only two long-term colonized Central and South American malaria vectors maintained in laboratories that have been used for experimental infections mostly by feeding them on blood of patients from endemic regions infected with *P*. *vivax*, demonstrating that they can be good models to study the interaction of American vectors with *Plasmodium* species [8]. More recently, *An*. *darlingi* has been colonized and successfully infected with *P*. *vivax* [9].

Studies in *An*. *gambiae* infected with *Plasmodium berghei*, a murine malaria model, revealed that the mosquito complement-like system can greatly limit *Plasmodium* infection [10]. The Thioester-containing Protein TEP1 is a major effector molecule that is stabilized in the mosquito hemolymph by interacting with two leucine-rich proteins, the leucine-rich repeat immune protein 1 (LRIM1) and the *Anopheles Plasmodium*-responsive leucine-rich repeat 1 protein (APL1) [11–13]. LRIM1 silencing results in premature activation of TEP1 and disrupts complement-mediated mosquito antiplasmodial responses, greatly increasing *An*. *gambiae* infection with *P*. *berghei* [12, 13], and with some strains *P*. *falciparum* strains[14] [15].

The establishment of robust animal models has been key to our current understanding of the biology of malaria transmission and the mosquito responses to *Plasmodium* infection. Understanding the parasite/vector interactions that affect vectorial capacity is indispensable for the development of new drugs or vaccines to disrupt transmission. The most widely used laboratory models to study malaria transmission are the *in vitro* production of *P*. *falciparum* gametocytes, a human malaria parasite, and *in vivo* infection with two murine parasites, *P*. *berghei* and *P*. *yoelii*. The two mosquito species most widely studied are the African vector *An*. *gambiae*, and the Asian vector *An*. *stephensi*. Mosquitoes can differ widely in their susceptibility to infection with specific *Plasmodium* parasite species [16] and great differences have been documented even between parasite strains [17, 18]. *An*. *albimanus* can be infected by direct feeding on *P*. *berghei*-infected mice [19] but, in general, infections are much lower when compared to *An*. *gambiae* or *An*. *stpehensi*. *An*. *albimanus* is readily infected with a *P*. *falciparum* strain of Brazilian origin (7G8) but is highly resistant to infection with African and Asian strains [18].

The main goal of this study was to establish robust experimental models to study malaria transmission by two colonized New World vectors, *An*. *aquasalis* and *An*. *albimanus*. Their susceptibility to laboratorial infections with the human malaria parasite *P*. *falciparum* and the rodent parasites *P*. *berghei* and *P*. *yoelii* was investigated. The role of the mosquito complement-like system in their susceptibility to infections was also evaluated by silencing LRIM1. Our studies show that *Plasmodium yoelii nigeriensis* is a viable model system to study malaria transmission by New World vectors.

## Results

### *Plasmodium falciparum* infection in *An*. *aquasalis*

**Fig 1:**
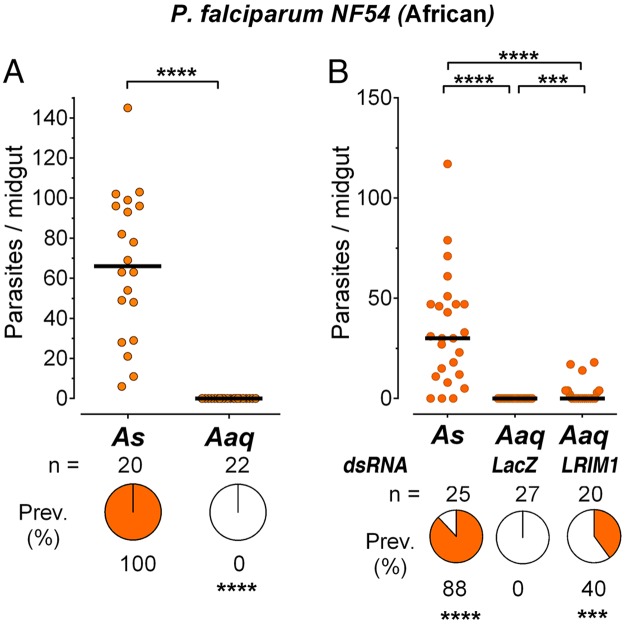
Infection of *A*. *aquasalis* with *P*. *falciparum* NF54. (A) Susceptibility of *Anopheles stephensi* (As) and *Anopheles aquasalis* (Aaq) mosquitoes to infection with *Plasmodium falciparum* NF54 strain. (B) Effect of disrupting the mosquito immune system by silencing LRIM1 on Aaq susceptibility to infection. Each dot represents the number of oocysts present on an individual midgut 10–12 days post-infection and the median number of oocysts is indicated by the black line. The medians were compared using the Mann-Whitney test and the infection prevalence using Chi-square (*** p<0.001, **** p<0.0001).

A major limitation in establishing experimental models for New World vectors is the impossibility of using mosquito vectors that are widely used elsewhere for laboratory infections (such as *An*. *gambiae* or *An*. *stephensi*) to ensure the quality of the gametocytes (positive controls) when performing experiments in laboratories located in malaria endemic areas. This problem was circumvented by transiently establishing an *An*. *aquasalis* colony at the National Institutes of Health (NIH) in the USA. The susceptibility of *An*. *aquasalis* to *P*. *falciparum* infection was evaluated using gametocyte cultures from the NF54 strain, a laboratory-adapted line of putative African origin. The quality of the gametocyte cultures was confirmed by simultaneously feeding *An*. *stephensi* (Nijmengen Sda500) mosquitoes, a laboratory strain that has been genetically selected to be highly susceptible to *P*. *falciparum* infection [20]. The *An*. *stephensi* control groups were readily infected with *P*. *falciparum* NF54, reaching infection prevalences of 88–100% and medians of 30–90 oocysts/midgut (Fig 1A and S1 Fig). In contrast, only one of the *An*. *aquasalis* mosquitoes out of 53 that fed on the same gametocyte cultures became infected with a single oocyst (p<0.0001, Fig 1A and S1 Fig).

We investigated whether the lack of infectivity of *An*. *aquasalis* was due to activation of the mosquito immune system by silencing LRIM1 expression. We were able to clone a 1,501bp partial sequence of the *An*. *aquasalis* LRIM1 cDNA (S2 Fig) using primers designed based on the annotated *An*. *albimanus* LRIM1 transcript (AALB005865-RA). This region has 81% identity to the *An*. *albimanus* LRIM1 transcript, and the 571bp 3’-end of our partial *A*. *aquasalis* cDNA has 100% homology to a shorter non-annotated transcript in Vector Base labeled GAMD01000377.1_Aaquasalis_Anoaqua-4332_mRNA. Our partial cDNA sequence (S2 Fig) was used to design primers to generate dsRNA and to evaluate the silencing efficiency.

None of the mosquitoes injected with the dsLacZ control were infected, while 40% of the *An*. *aquasalis* mosquitoes in which LRIM1 was silenced became infected (p<0.001, Fig 1B). However, even after silencing LRIM1 the infection intensity was still low (median of 0) in *An*. *aquasalis*, when compared to *An*. *stephensi* with median of 38 oocysts (p<0.0001), and a prevalence of 88% (p<0.001); indicating that only some of the parasites are eliminated by the *An*. *aquasalis* immune system.

**Fig 2:**
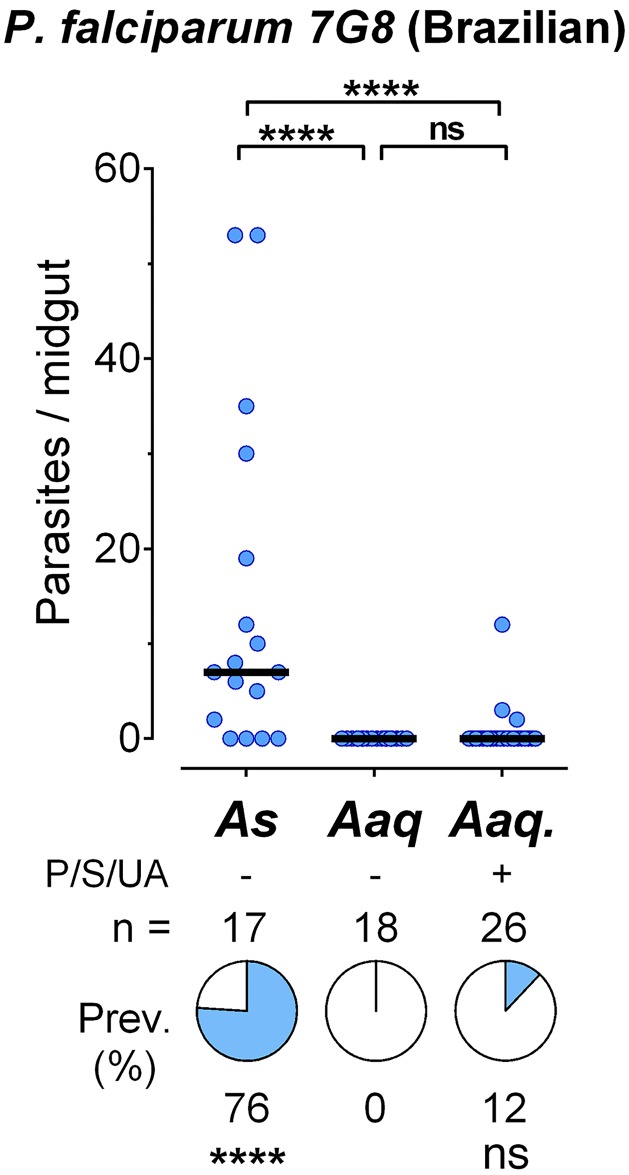
Infection of *A*. *aquasalis* with *P*. *falciparum* 7G8. Susceptibility of *Anopheles stephensi* (As) and *Anopheles aquasalis* (Aaq) mosquitoes to infection with *Plasmodium falciparum* 7G8 strain. The effect of oral administration of antibiotics (Penicillin/Streptomycin = P/S) and uric acid (UA) on Aaq infection was also tested. Each dot represents the number of oocysts present on an individual midgut 10–12 days post-infection and the median number of oocysts is indicated by the black line. The medians were compared using the Mann-Whitney test and the infection prevalence using Chi-square (**** p<0.0001).

Recent studies have shown that parasite isolates from different geographic origin can exhibit dramatic differences in infectivity to the same mosquito vector. For example, a *P*. *falciparum* line of Brazilian origin (7G8) is more effective infecting *An*. *albimanus* mosquitoes than parasite lines of African origin [18]. It is also well established that oxidative stress [21] and the gut microbiota [22] can affect *Plasmodium* survival. Oral administration of uric acid reduces oxidative stress, decreasing loss of fecundity with age and preventing *Plasmodium* melanization [21, 23]. We tested the susceptibility of *An*. *aquasalis* to infection with the *P*. *falciparum* 7G8 line and the effect of reducing the gut microbiota by oral administration of antibiotics solution (Penicillin and Streptomycin). The solution was also supplemented with uric acid, to reduce oxidative stress in the mosquito. Although a few *An*. *aquasalis* subjected to this treatment became infected with *P*. *falciparum* 7G8 (Fig 2 and S3 Fig), the prevalence was low (10–12%) when compared to *An*. *stephensi* (28–76%, p<0.001). None of the *An*. *aquasalis* females that were not treated with the antibiotic + uric acid mixture became infected (p<0.0001).

### Susceptibility of *An*. *aquasalis* to infection with murine malaria parasites

**Fig 3:**
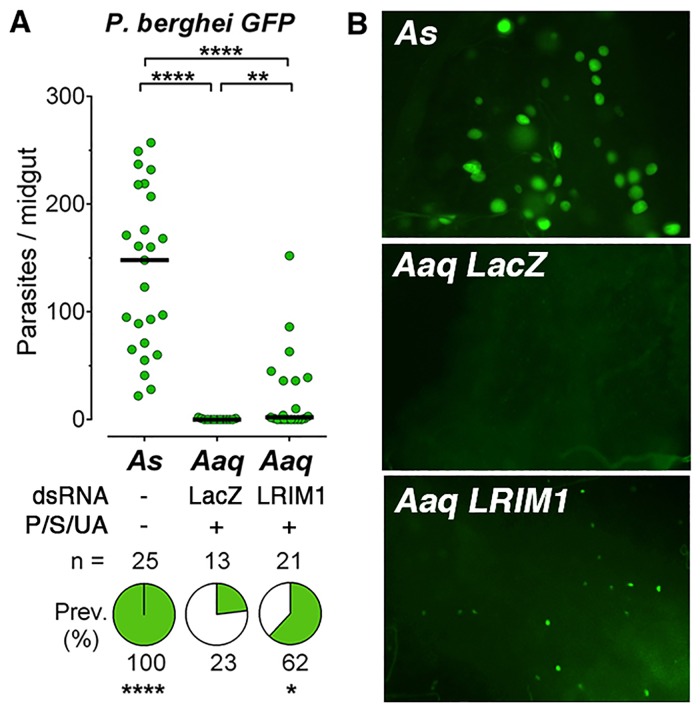
Infection of *A*. *aquasalis* with *P*. *berghei*. Susceptibility of *Anopheles stephensi* (As) and *Anopheles aquasalis* (Aaq) mosquitoes to infection with *P*. *berghei*. (A) Effect of disrupting the mosquito immune system by silencing LRIM1 on Aaq susceptibility to infection. (B) *P*. *berghei* oocysts in As and Aaq mosquitoes 8 days post infection. Each dot represents the number of oocysts present on an individual midgut 10–12 days post-infection and the median number of oocysts is indicated by the black line. The medians were compared using the Mann-Whitney test and the infection prevalence using Chi-square (* p<0.05, **** p<0.0001).

The *P*. *berghei* ANKA 2.34 strain can effectively infect *An*. *albimanus* when mosquitoes are fed ookinetes cultured *in vitro*, and a high prevalence (>90%) and intensity of infection (20–30 oocysts/midgut) can be obtained [19]. Multiple times we attempted to infect females from a *An*. *aquasalis* colony established in Brazil by direct feeding on *P*. *berghei* (Anka 2.34-GFP)-infected mice with no success, even when mice parasitemias and exflagellations were optimal (data not shown). We decided to confirm this lack of infectivity at the Laboratory of Malaria and Vector Research (NIH), by infecting *An*. *stephensi* and *An*. *aquasalis* using the same mouse, under optimal conditions. As expected, the *An*. *stephensi* control group was very susceptible to infection with *P*. *berghei* (Anka 2.34-GFP) with a prevalence of 100% and median of 148 oocysts 7 days post-feeding. In contrast, only three of the *An*. *aquasalis* females treated with the antibiotic + uric acid solution and injected with a dsLacZ control dsRNA became infected with a single oocyst (p<0.0001, Fig 3A). Silencing LRIM1 significantly enhanced the infection prevalence to 62% (p<0.04), but the intensity of infection remained low (median of 1) (p<0.01, Fig 3A). Furthermore, the oocysts that developed were very small at 7 days post-feeding, relative to those in *A*. *stephensi*, (Fig 3B) indicating that the ookinetes that survived in *An*. *aquasalis* did not develop normally into the oocysts stage.

**Fig 4:**
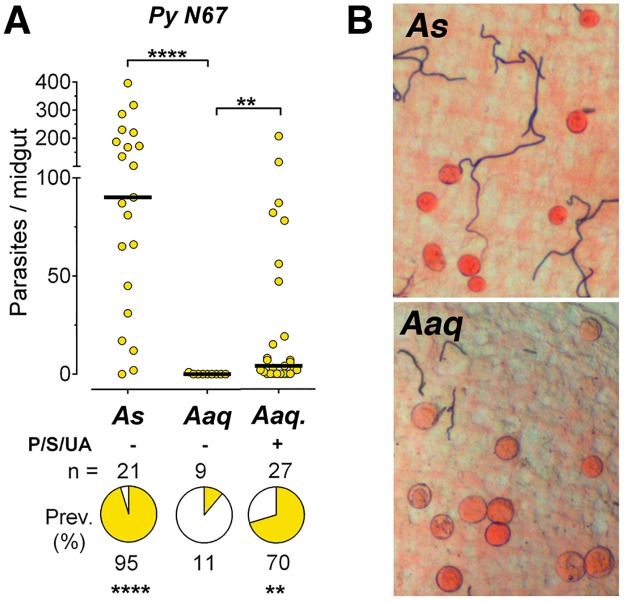
Infection of *A*. *aquasalis* with *P*. *yoelii nigeriensis N67*. Susceptibility of *Anopheles stephensi* (*As*) and *Anopheles aquasalis* (*Aaq*) mosquitoes to infection with *P*. *yoelii nigeriensis N67* (PyN67). (A) Effect of oral administration of antibiotics (Penicillin/Streptomycin = P/S) and uric acid (UA) on *Aaq* infection. (B) PyN67 oocysts in *As* and *Aaq* mosquitoes 8 days post infection. Image of oocysts in *Aaq* mosquitoes treated with P/D + UA. Each dot represents the number of oocysts present on an individual midgut 10–12 days post-infection and the median number of oocysts is indicated by the black line. The medians were compared using the Mann-Whitney test and the infection prevalence using Chi-square (** p<0.01, **** p<0.0001).

**Fig 5:**
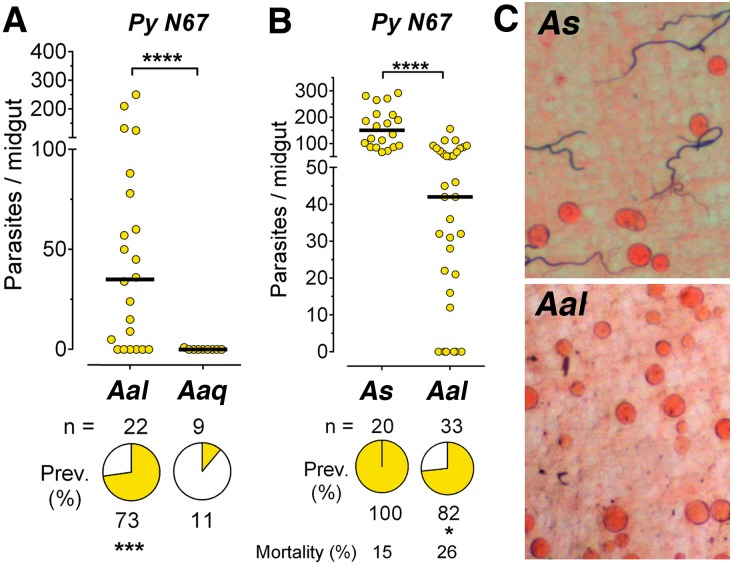
Infection of *A*. *albimanus* with *P*. *yoelii nigeriensis N67*. (A and B) Susceptibility of *Anopheles albimanus* (Aal), *Anopheles aquasalis* (Aaq) and *Anopheles stephensi* (As) mosquitoes to infection with *P*. *yoelii nigeriensis N67* (PyN67), without antibiotics or uric acid. (C) PyN67 oocysts in *As* and *Aal* mosquitoes 8 days post infection. Each dot represents the number of oocysts present on an individual midgut 10–12 days post-infection and the median number of oocysts is indicated by the black line. The medians were compared using the Mann-Whitney test and the infection prevalence using Chi-square (* p<0.05, *** p<0.001, **** p<0.0001).

Given the extremely low infectivity of *P*. *berghei*, we decided to test a different murine malaria parasite. We found that *An*. *aquasalis* was much more susceptible to infection with *P*. *yoelii nigeriensis* N67 (PyN67) parasites, reaching a consistent high prevalence of infection (54–70%) when mosquitoes were treated with antibiotic + uric acid solution (p<0.01), (Figs 4A and 5A, and S4 Fig). In contrast to *P*. *berghei*, PyN67 oocysts developed normally in *An*. *aquasalis* and had a similar size and appearance as in *An*. *stephensi* controls (Fig 4B). We were able to recover sporozoites from the salivary glands of *An*. *aquasalis* (17,000–19,000 spz/mosquito) indicating that PyN67 can complete its life cycle in this mosquito species. The infectivity of these sporozoites to mice was not tested.

### Susceptibility of *An*. *albimanus* to infection with *P*. *yoelii* N67

**Fig 6:**
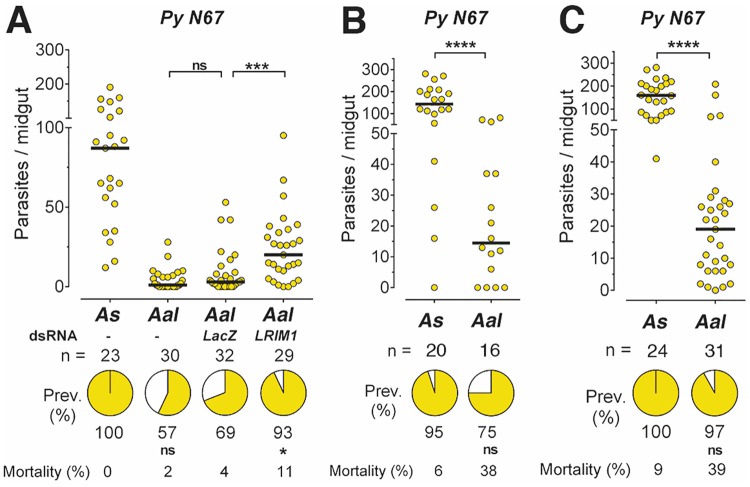
Effect of disrupting the mosquito immune system on *PyN67* infection in *A*. *albimanus*. Susceptibility of *Anopheles stephensi* (*As*) and *Anopheles albimanus* (*Aal*) mosquitoes to infection with *P*. *yoelii nigeriensis N67* (PyN67). (A) Susceptibiltiy to infection and effect of disrupting the mosquito immune system by silencing LRIM1. (B and C) Susceptibility of infection and mortality after PyN67 completed one developmental cycle in *An*. *albimanus*. Each dot represents the number of oocysts present on an individual midgut 10–12 days post-infection and the median number of oocysts is indicated by the black line. The medians were compared using the Mann-Whitney test and the infection prevalence using Chi-square (*p<0.05, *** p<0.001, **** p<0.0001, ns = not significant).

*An*. *albimanus* is an important malaria vector in Mexico, Central America and the Northern regions of South America. We explored the potential of *P*. *yoelii* N67 as a model of malaria transmission in this mosquito species. *An*. *albimanus* is more susceptible to infection with PyN67 than *An*. *aquasalis* (Fig 5A), reaching a high prevalence (73%) of infection and a median of 36 oocysts/midgut without administration of antibiotics or uric acid, while no oocysts could be detected in *An*. *aquasalis* females (p<0.0001, Fig 5A). However, the intensity and prevalence of infection was significantly lower than in *An*. *stephensi* (p<0.0001, Fig 5B and S5 Fig). PyN67 oocysts also developed normally in *An*. *albimanus* (Fig 5C). Injection of dsLacZ control dsRNA had no effect, while silencing LRIM1 significantly increased the intensity of infection (p<0.001, Fig 6A and S6 Fig), indicating that the *An*. *albimanus* complement-like system limits PyN67 infection.

We were able to recover a small number of sporozoites from infected salivary glands of *An*. *albimanus* mosquitoes (200–500 spz/mosquito), and they were used to infect a recipient mouse. The infection was successful when 1000 PyN67 sporozoites extracted from *A*. *albimanus* salivary glands were injected IV into a BalbC mouse, indicating that PyN67 parasites can complete their development in *An*. *albimanus* and generate infectious sporozoites. In general, after completing one developmental cycle in *An*. *albimanus*, mosquito infections were high, often reaching medians of more than 100 oocysts in *An*. *stephensi* (Fig 6B and 6C and S7 Fig). Interestingly, these parasites seemed to be more pathogenic to *An*. *albimanus* mosquitoes, as they caused 6–9% mortality in *A*. *stephensi*, while in *An*. *albimanus* mortality was significantly higher 20–39% (p<0.01).

## Discussion

*An*. *aquasalis* is susceptible to infection with *P*. *vivax* [24] and this experimental system has been used to investigate the role of the STAT pathway [25] and reactive oxygen species [26] on mosquito susceptibility to infection. Recent studies indicate that *P*. *falciparum* evasion of mosquito immunity is mediated by the *Pfs47* gene and is critical for parasite survival. Different haplotypes of *Pfs47* are circulating in different continents and they are major determinants of vector/parasite compatibility [18]. *An*. *gambiae* mosquitoes are very susceptible to infection with two different *P*. *falciparum* lines of African origin (NF54 and MRA1181), while *An*. *albimanus* is highly refractory to infection with these isolates. Conversely, *An*. *albimanus* is more susceptible to infection with a *P*. *falciparum* line (7G8) of Brazilian origin than *An*. *gambiae* [18]. The lack of compatibility between isolates from a different continent can be overcome by disrupting the mosquito complement-like system, indicating that the mosquito immune system is selecting for parasites that express certain haplotypes of *Pfs47* and can evade immunity [18]. We found that *An*. *aquasalis* is almost completely refractory to infection with *P*. *falciparum* NF54 parasites, however, although disrupting the mosquito complement-like system by silencing LRIM1 significantly increased the prevalence and intensity of infection, the infection levels are much lower than in *An*. *stephensi* controls or in *An*. *albimanus* females in which LRIM1 expression was silenced [18]. This suggests that, besides the mosquito immune system, some other factor(s) in *An*. *aquasalis* is probably responsible for the low infectivity with *P*. *falciparum* NF54 parasites. This is in agreement with the observation that *An*. *aquasalis* is also highly refractory to infection with a *P*. *falciparum* (7G8) line expressing the most common *Pfs47* haplotype in Brazil. Oral administration of antibiotics and uric acid allowed survival of a few parasites, but the prevalence and intensity of infection were still low. While it is clear that *An*. *aquasalis* is an important vector of *P*. *vivax* malaria in Brazil (reviewed by [8]) and Guyana [27], our findings indicate that this mosquito species is not a competent vector of *P*. *falciparum* malaria with the two different lines tested. In the Amazon region, *An*. *darling*, *An*. *albitarsis* and *An*. *rondoni* have been documented as vectors of *P*. *falciparum* by direct immunodetection of sporozites, with *An*. *darling* being the most prevalent infected species. This kind of direct evidence for *An*. *aquasalis* as a major vector of *P*. *falciparum* malaria is not available.

*An*. *aquasalis* is less susceptible to infection with *P*. *berghei* and *P*. *yoelii* N67 than *An*. *albimanus*. This could be due to differences in physiology or in the microbiota, as *An*. *aquasalis* larvae have adapted to brackish water. Reducing the microbiota by oral administration of antibiotics and disruption of the *An*. *aquasalis* immune system was able to rescue some low level of *P*. *berghei* infection, indicating that a few ookinetes were successful in invading the midgut and transformed into oocyts. However, the oocysts that formed were very small, indicating that they did not develop properly (Fig 3B). This could be due to physiological conditions in *An*. *aquasalis* that do not provide and adequate environment for the developing oocysts or to late phase immune responses that target the oocyst stage of the parasite. *An*. *aquasalis* females treated with antibiotics and uric acid were much more susceptible to infection with PyN67 than with *P*. *berghei* and the oocysts that developed were normal in size; indicating that murine PyN67 infection is a good animal model to study malaria transmission by *An*. *aquasalis*.

*An*. *albimanus* is more susceptible to PyN67 infection than *An*. *aquasalis*, and it is readily infected without the need for oral administration of antibiotics or uric acid. It also supports normal oocyst development. Silencing the *An*. *albimanus* immune system enhances infection, indicating that mosquitoes are mounting an active immune response to infection. Furthermore, PyN67 salivary gland sporozoites were infectious to mice, making it possible to close the transmission cycle under laboratory conditions. In all experiments, *An*. *stephensi* was more susceptible to infection than *An*. *aquasalis* and *An*. *albimanus*, probably because this line was genetically selected to be highly susceptible to *P*. *falciparum* infection and these mosquitoes take very large blood meals under laboratory conditions. We conclude that different anopheline mosquito species differ broadly in their susceptibility to infection with different *Plasmodium* species. *P*. *yoelii* N67 appears to have a broad ability to infect many different mosquito species, including New World vectors, making it an excellent model system to study malaria transmission.

## Materials and Methods

### Ethics statement

Public Health Service Animal Welfare Assurance #A4149-01 guidelines were followed according to the National Institutes of Health Animal (NIH) Office of Animal Care and Use (OACU). These studies were done according to the NIH animal study protocol (ASP) approved by the NIH Animal Care and User Committee (ACUC), with approval ID ASP-LMVR5. All the animal procedures used in this study have been approved with the NIH Animal Care and User Committee (ACUC).

### Mosquito rearing

*An*. *Stephensi*, *Anopheles aquasalis* and *Anopheles albimanus* mosquitoes were reared at 27°C and 80% humidity on a 12-h light/dark cycle under insectary conditions [21]. All mosquito larvae were readed in unchlorinated water, by allowing chorinated water to rest for 48h in an open container. Tetramin Tropical Flakes^®^ fish food was grinded into fine powder using a coffee mill and used as feeding source for larvae. *An*. *aquasalis* larvae were reared in water that contained table salt at a final concentration of 2g/L. *An*. *aquasalis* adult females were fed on cow blood for colony maintenance by membrane feeding (using hog gut sausage casings as membranes), while *An*. *albimanus* and *An*. *stephensi* females were fed on live anesthetized chickens.

Mosquitoes were fed a 10% sucrose solution in a cotton ball until two days before the infective blood meals, when some experimental groups were switched to drinking a 1% uric acid solution with Penicillin (100 units/ml) and Streptomycin (0.1 mg/ml) in water, and they obtained sugar from a solid sugar cube placed on top of the cage. They were fed this solution and the sugar cube until the end of the experimental infections. The solution was fed by placing it in an inverted glass test tube with a cotton ball plug and was changed daily.

### *Plasmodium* infections

Female mosquitoes (4–5 days old) were infected by feeding them blood meals with mature stage IV and V *P*. *falciparum* gametocyte cultures (NF54 or 7G8 strains) through a membrane feeder at 37°C for 30 min [17]. Human blood was obtained from the Interstate Blood Bank, Memphis, Tennessee. Membrane feedings were done using hog gut sausage casings as membranes.

For *P*. *yoelii nigeriensis* (N67 strain) and *P*. *berghei* infections, parasites from frozen stocks were administered via intraperitoneal (IP) injection to 3- to 5-week-old BALB/c female mice. When the parasitemia of the donor mice reached 5–10% (in about 5–7 days), the infected blood was taken by cardiac puncture and transferred to a healthy mouse. Mouse parasitemia was determined by light microscopy inspection of Giemsa-stained thin blood smears obtained by tail snip. Experimental BALB/c mice were infected by intraperitonealy (IP) injection 20–30μl fresh blood from the donor mice. This recipient mouse was used to infect mosquitoes when it reached 3–5% parasitemia 2–3 days after inoculation. Female mosquitoes (5–7 days old) were infected by direct feeding on anesthetized infected mice. For *P yoelii* infections, mosquitoes were maintained at 24°C, while *P*. *berghei*-infected mosquitoes were kept at 21°C (their respective permissive temperatures for gametogenesis). Both were kept at 80% humidity. *P*. *yoelii* midgut infection was assessed by light microscopy 10–11 days after feeding with mercurochrome staining (0.1% in water) [28]; while *P*. *berghei* midgut infection was assessed by fluorescence microscopy of GFP parasites 7 days after feeding. Oocysts on individual midguts were counted to determine the prevalence and intensity of infection.

The number of PyN67 sporozoites recovered from *An*. *aquasalis* salivary glands was between 17,000–19,000 spz/mosquito. Their infectivity to mice was not tested. The number of sporozoites recovered from *An*. *albimanus* salivary glands was between 200–500 spz/mosquito. We injected 1000 *P*. *yoelii* N67 sporozoites extracted from *An*. *albimanus* salivary glands via IV into the tail vein of a Balb/c mouse in a volume of 100 μl of RPMI with 10% mouse serum, and the animal became infected.

### RNAi gene silencing

Individual female *A*. *gambiae* mosquitoes were injected 1–2 d after emergence as previously described [21]. Briefly, mosquitoes were injected with 69 nL of a 3 μg/μL dsRNA solution 3–4 d before receiving a Plasmodium-infected blood meal. The control dsRNA (dsLacZ) was produced as previously described [21]. Double-stranded dsLacZ RNA was generated by introducing T7 promoters (in bold letters) thought PCR amplification of the cloned insert using the following vector primers: M13F 5' GTAAAACGACGGCCAG 3' and M13Rev- T7 5'CTCGAG**TAATACGACTCACTATAGGG**CAGGAAACAGCTATGAC3'. The PCR product was used as template to generate dsRNA using T7 RNA polymerase, as described below.

The *An*. *aquasalis* LRIM1 dsRNA was synthesized with primers designed based on the partial An. aquasialis LRIM1 cDNA sequence (S2 Fig). The sequence of the primers used is: LRIM1_AaqFw, 5’- **TAATACGACTCACTATAGGG**TTGTACGGCACGGTAAACCT-3’, and LRIM1_AaqRv, 5’-**TAATACGACTCACTATAGGG**CCACGGTAGCTTGTTGTGC-3’ (PCR conditions were 94°C for 3min; 40 cycles of 94°C for 30 s, 59°C for 30 s, and 72°C for 1min; final extension, 72°C, 5 min).

The primers used for *An*. *albimanus* LRIM1 were as follows: two external PCR primers were used, and the product of the first amplification was used as template for a second one using internal primers with the following sequences:

LRIM1_AalExFw, 5’-AAGGTTGAGCCGAAGAATGA-3’, andLRIM1_AalExRv,5’-GCACTTCCCATGCTGCTAAT-3’ (PCR conditions were 94°C for 3min; 35 cycles of 94°C for 30 s, 55°C for 30 s, and 72°C for 1 min; final extension, 72°C, 5 min); for internal PCR (primers containing T7 promoter): LRIM1_AalInFw, 5’-**TAATACGACTCACTATAGGG**CTGTACGGCACCGTTAACCT-3’, and LRIM1_AalInRv, 5’-**TAATACGACTCACTATAGGG**AGCTTGTTGTGCGAAAGGTC-3’ (PCR conditions were 94°C for 3min; 40 cycles of 94°C for 30 s, 59°C for 30 s, and 72°C for 1min; final extension, 72°C, 5 min; using 1 μL of a 1–20 dilution of the external primer PCR).

RNAs were synthesized simultaneously from the template, annealed and purified using the T7 RNAi Mega-script kit (Ambion) following the procedure recommended by the manufacturer. dsRNA products were eluted in water to a final concentration of 3 μg/μl. A volume of 69nl of dsRNA preparation was injected into the thorax of cold anesthetized, 2–3 day-old female mosquitoes using a nano-injector (Nanoject; Drummond Scientific, Broomall, Pennsylvania, USA) fitted with a glass capillary needle. The dsLacZ RNA was used in each experiment to control for any unspecific effect of dsRNA injection. Mosquitoes received a *Plasmodium*-infected blood meal 2–3 day post-dsRNA injection.

### qRT-PCR gene expression

Total RNA was isolated from 15 to 20 mosquito midguts using Trizol (Invitrogen) and cDNA synthesis was performed using QuantiTect Reverse Transcription Kit (Qiagen). Gene-expression analysis was measured by SYBR green qRT-PCR (DyNAmo HS; New England Biolabs) in a CFX96 system (Biorad). Gene expression was assessed using two to three technical replicates and three biological replicates. The *An*. *gambiae* ribosomal protein S7 was used as an internal reference to normalize each sample for the amount of template present. Fold-change was calculated using the 2−ΔΔ Ct method.

*An*. *aquasalis* and *An*. *albimanus* LRIM1 gene silencing was assessed in whole sugar-fed mosquitoes by quantitative real-time PCR (qPCR) using the S7 ribosomal protein gene as internal reference. The primers used for qPCR for *A*. *aquasalis* were as follows: LRIM1_Aaq_qPFw, 5’-ACCTCAGCGGTAACAAGGTG-3’; LRIM1_Aaq_qPRv; 5’- CTGCGGGTCCTTATTGTTTG-3’; S7_Aaq_qPFw, 5’- ATCCTGGAGCTGGAGATGAA -3’; and S7_Aaq_qPRv, 5’- ACGATGGCCTTCTTGTTGTT -3’.

The primers used for qPCR for *A*. *albimanus* were as follows: LRIM1_Aal_qPFw, 5’-GACAAAAGTGTGCGCTTTGA-3’; LRIM1_Aal_qPRv; 5’-CACTCCCGGATTAGAGCTTG-3’; S7_Aal_qPFw, 5’-ACCTGGACAAGAACCAGCAG-3’; and S7_Aal_qPRv, 5’-GTTTTCTGGGAATTCGAACG-3’. The silencing efficiency in dsRNA-injected mosquitoes was 94–98% for *An*. *aquasalis* LRIM1 and 85–98% for *An*. *albimanus* LRIM1, relative to dsLacZ-injected controls.

### Statistical analysis

All statistical analyses were performed using GraphPad Prism 5 (GraphPad software). Analyses derived from at least two independent biological replicates. Gene-expression data were analyzed using Student’s t test on mean value of all independent experiments. Infection intensity of oocysts was compared with each other using Mann–Whitney tests, infection prevalence and overall mortality were compared using χ^2^ analysis.

## Supporting Information

S1 Fig Susceptibility of *Anopheles stephensi* (As) and *Anopheles aquasalis* (Aaq) mosquitoes to infection with *Plasmodium falciparum* NF54 strain. (DOCX)

S2 Fig Partial nucleotide sequence of *An*. *aquasalis* LRIM1 cDNA. (DOCX)

S3 Fig Susceptibility of *Anopheles stephensi* (As) and *Anopheles aquasalis* (Aaq) mosquitoes to infection with *Plasmodium falciparum* 7G8 strain. (DOCX)

S4 Fig Susceptibility of *Anopheles stephensi* (As) and *Anopheles aquasalis* (Aaq) mosquitoes to infection with *P*. *yoelii nigeriensis N67* (PyN67). (DOCX)

S5 Fig Susceptibility of *Anopheles stephensi* (*As*) and *Anopheles albimanus* (*Aal*) to infection with *P*. *yoelii nigeriensis N67* (PyN67), without antibiotics or uric acid. (DOCX)

S6 Fig Susceptibility of *Anopheles stephensi* (*As*) and *Anopheles albimanus* (*Aal*) mosquitoes to infection with *P*. *yoelii nigeriensis N67* (PyN67), and effect of disrupting the mosquito immune system by silencing LRIM1 on *Aal* susceptibility to infection. (DOCX)

S7 Fig Susceptibility of *Anopheles stephensi* (*As*) and *Anopheles albimanus* (*Aal*) mosquitoes to infection with *P*. *yoelii nigeriensis N67* (PyN67) and mortality after PyN67 completed one developmental cycle in *An*. *albimanus*. (DOCX)

## Abbreviations

APL1: *Anopheles Plasmodium*-responsive leucine-rich repeat 1 protein
LRIM1: leucine-rich repeat immune protein 1
TEP1: Thioester-containing Protein
