# Supplementary material for: Plasmodium yoelii nigeriensis (N67) Is a Robust Animal Model to Study Malaria Transmission by South American Anopheline Mosquitoes
Source: PLoS One. 2016 Dec 2;11(12):e0167178. doi: 10.1371/journal.pone.0167178 (PMC5135088; doi:10.1371/journal.pone.0167178)
Supplement: S2 Fig — (DOCX) [file pone.0167178.s002.docx]

**S2 Figure.**  **Partial nucleotide sequence of *An. aquasalis* LRIM1 cDNA.**

>Aquasalis_LRIM1_Partial cDNA

GGACAACAGCCGCACCTTTGGTGACTTCCAGCTAACGTTGAAAATGGCATTACTTTGGTCGTGCTCACTAGCTACGCTGCGGGAGCGTTCTTTAAGGTTGAGCCGAAAAATGATGGCAGACCGGCATCCTACAAGATCAGCCAAGTTACCAGCCTCAATTTGGGACAAGCAATGCTTCAACTTCCTCACAACGTACAGGCGCTTGATTTGAGTGGCAACTTGCTGACCAACATAAGTGGTAGTGCCTTTAGTGCGTTCTCCAACTTGCAGCAGCTGAATCTATCATCCAATATGTTGTACGGCACGGTAAACCTGACTCAGTTTTACACTCTCAGCACGGTCGATTTGAACGACAACTACATCACCGATCTGCATCTTGGACGAAATGTGGCAACAGTATCAGCCGTTCGTAATCGTATCAAGCGAGTAATCTGTGACGGATCGCCGAAGGAAGTGAATTTGCAGGTGAACAAGGTGGACTCACTACATGGCCTGTCACCAATGTGCATGACAAGATTGCAGGTTTTGGATTTATCCTTAAATGAGGTGAATACGATCGATTTTAATTATCTGGCGCCTTCCGCAAACTCGTTGAAGCAATTGCTGTTATCGTACAACTACATATACGAACTGTTTAACAAGAACAACCTTGTGTTCCCGCTTCTCGAGGTGCTCGATCTATCGCACAACAAGCTACCGTGGCTTAGTCCAGACATAATGGTCGCCCGGAATGCAAAAACCGTCGATCTATCGGCTAACCAAATTGTTTTGATTGACAAAAGTATCCAGTTCGATCGGCAAACTAATATCAACCTCAGCGGTAACAAGGTGCAGTGTGAATCGTTAAAAGCGTTCGCAACATTAAATCCAGCAGTGAAGAATGTGAGTCCGGCAAACAATAAGGACCCGCAGGGATGCAACCGGATGTCTGGTTATTCGATCTGCTGTGATTCATTGTCAGCACCGTTCGCGGACCGACTGATCGAGCAGAAGCGCATGCAAAACTCGCTGCTCAATGTACCGATGGGTCCGGGGGCGAAACCAAACTGTACCGTCGATGATGCTCGGCAGACAATGATTAGCCAAATGGGTAGTGCGATCACATCGGTCGCTAATGAGGTACAGCGGCTACAGAAGGAGAAGATCCAGCTTGCTTCCGAGCGCCAGGCTCTGGAGCAGACGGTCAGTGCACAGAGGGAACAAAGTACCAGTGTGCGTGAGGCTCTGCTTGCAGCTGCGCGAAAATTGAACCTTCAAGTTGAGCAGGAACCGAGCCACGTCGTTTTGCAGAAGGTGATCGATACGTACGAACATCTGAGCAAGCAGGAGGAGCTGGAACGGAACAAAGCCACCGAGGACTGGAACAAGTACAGTACGGAGATTGAGCATTGGCTAAAGGAGAAGGAGCGCCTGGAGCCGCTCATCGCAAAGTATGACGCGGACATAAGCAAGGCCAACGCCACGCTGGTGGATCTCACCAGACAGAAGGCGGTTCTGACCG
