# Supplementary material for: Plasmodium yoelii nigeriensis (N67) Is a Robust Animal Model to Study Malaria Transmission by South American Anopheline Mosquitoes
Source: PLoS One. 2016 Dec 2;11(12):e0167178. doi: 10.1371/journal.pone.0167178 (PMC5135088; doi:10.1371/journal.pone.0167178)
Supplement: S4 Fig — (DOCX) [file pone.0167178.s004.docx]

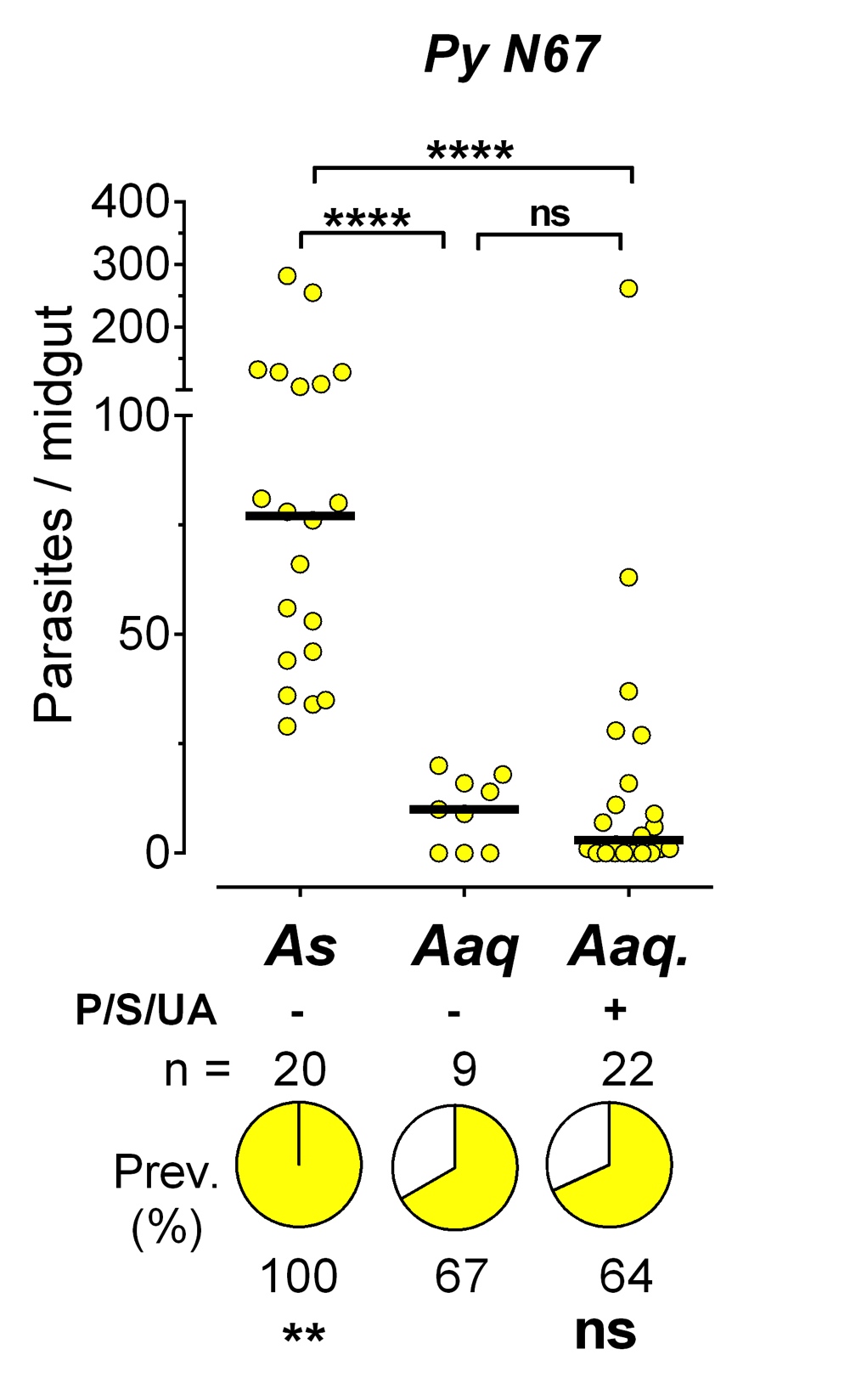


**S4 Figure.**  **Susceptibility of *Anopheles stephensi* (As) and *Anopheles aquasalis* (Aaq) mosquitoes to infection with *P. yoelii nigeriensis N67* (PyN67).** Effect of oral administration of antibiotics (Penicillin/Streptomycin = P/S) and uric acid (UA) on Aaq infection. PyN67 oocysts in As and Aaq mosquitoes 8 days post-infection. Each dot represents the number of oocysts present on an individual midgut 10-12 days post-infection and the median number of oocysts is indicated by the black line. The medians were compared using the Mann-Whitney test and the infection prevalence using Chi-square (**** p<0.0001, ns=not significant).
