# Supplementary material for: Plasmodium yoelii nigeriensis (N67) Is a Robust Animal Model to Study Malaria Transmission by South American Anopheline Mosquitoes
Source: PLoS One. 2016 Dec 2;11(12):e0167178. doi: 10.1371/journal.pone.0167178 (PMC5135088; doi:10.1371/journal.pone.0167178)
Supplement: S5 Fig — (DOCX) [file pone.0167178.s005.docx]

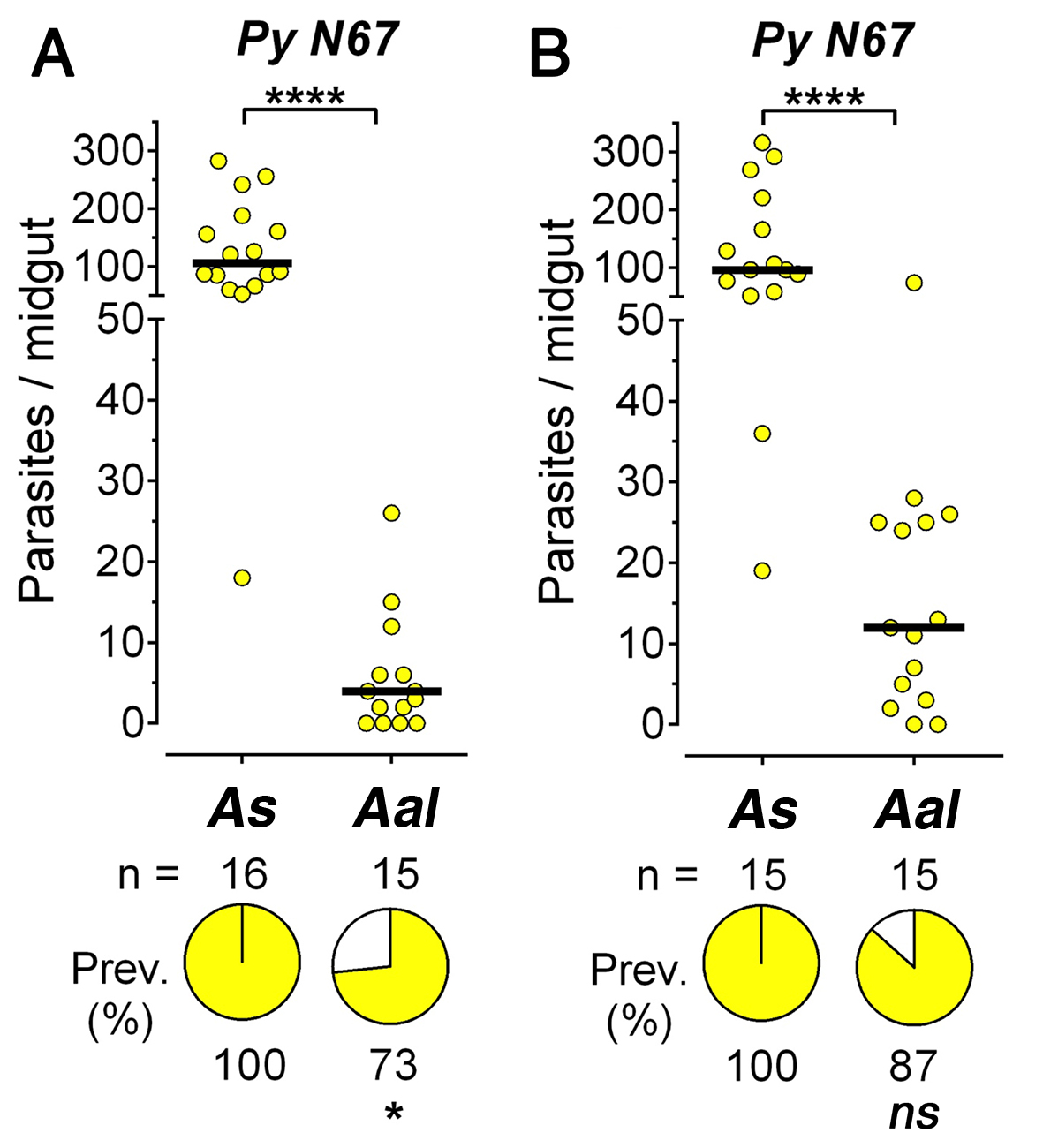


**S5 Figure.**  **Susceptibility of *Anopheles stephensi* (*As*) and *Anopheles albimanus* (*Aal*) to infection with *P. yoelii nigeriensis N67* (PyN67), without antibiotics or uric acid.** Each dot represents the number of oocysts present on an individual midgut 10-12 days post-infection and the median number of oocysts is indicated by the black line. Panesl A and B represent two independent experiments. The medians were compared using the Mann-Whitney test and the infection prevalence using Chi-square (ns= not significant, * p<0.05, **** p<0.0001).
