# Supplementary material for: Plasmodium yoelii nigeriensis (N67) Is a Robust Animal Model to Study Malaria Transmission by South American Anopheline Mosquitoes
Source: PLoS One. 2016 Dec 2;11(12):e0167178. doi: 10.1371/journal.pone.0167178 (PMC5135088; doi:10.1371/journal.pone.0167178)
Supplement: S7 Fig — (DOCX) [file pone.0167178.s007.docx]

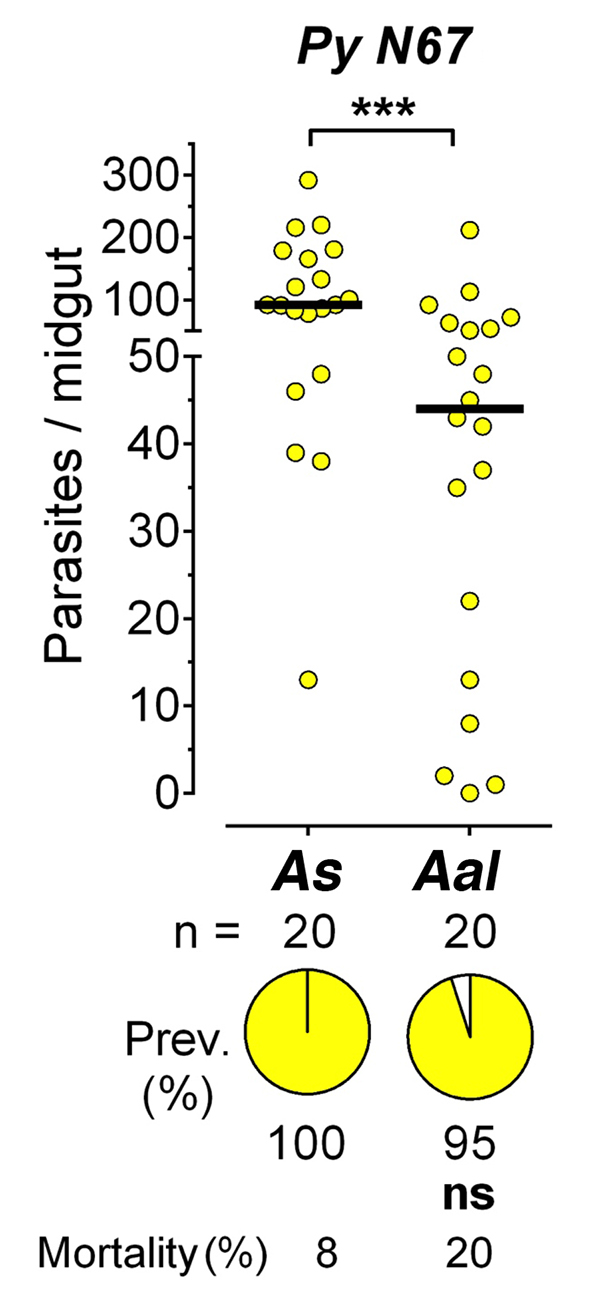


**S7 Figure. Susceptibility of *Anopheles stephensi* (*As*) and *Anopheles albimanus* (*Aal*) mosquitoes to infection with *P. yoelii nigeriensis N67* (PyN67) and mortality after PyN67 completed one developmental cycle in *An. albimanus*.** Each dot represents the number of oocysts present on an individual midgut 10-12 days post-infection and the median number of oocysts is indicated by the black line. The medians were compared using the Mann-Whitney test and the infection prevalence using Chi-square (*** p<0.001, ns = not significant).
